# Caloric Restriction (CR) Plus High-Nitrate Beetroot Juice Does Not Amplify CR-Induced Metabolic Adaptation and Improves Vascular and Cognitive Functions in Overweight Adults: A 14-Day Pilot Randomised Trial

**DOI:** 10.3390/nu15040890

**Published:** 2023-02-10

**Authors:** Mushari Alharbi, Martina Chiurazzi, Gilda Nasti, Espedita Muscariello, Teresa Mastantuono, Christina Koechl, Blossom CM Stephan, Oliver M Shannon, Antonio Colantuoni, Mario Siervo

**Affiliations:** 1 School of Life Sciences, The University of Nottingham Medical School, Queen’s Medical Centre, Nottingham NG7 2UH, UK; 2 Department of Clinical Biochemistry, Faculty of Medicine, King Abdulaziz University, Jeddah 22252, Saudi Arabia; 3 Department of Clinical Medicine and Surgery, Federico II University of Naples, Via S. Pansini, 5, 80131 Naples, Italy; 4 Human Nutrition Research Centre, Population Health Sciences Institute, Newcastle University, Newcastle upon Tyne NE2 4HH, UK; 5 Institute of Mental Health, The University of Nottingham Medical School, Nottingham NG7 2UH, UK; 6 Curtin School of Population Health, Curtin University, Kent Street, Bentley, WA 6102, Australia

**Keywords:** dietary nitrate, nitric oxide, caloric restriction, cognitive function, endothelial function, obesity

## Abstract

Caloric restriction (CR) and dietary nitrate supplementation are nutritional interventions with pleiotropic physiological functions. This pilot study investigates the combined effects of CR and nitrate-rich beetroot juice (BRJ) on metabolic, vascular, and cognitive functions in overweight and obese middle-aged and older adults. This was a two-arm, parallel randomized clinical trial including 29 participants allocated to CR + BRJ (*n* = 15) or CR alone (*n* = 14) for 14 days. Body composition, resting energy expenditure (REE), and hand-grip strength were measured. Resting blood pressure (BP) and microvascular endothelial function were measured, and Trail-Making Test A and B were used to assess cognitive function. Salivary nitrate and nitrite, and urinary nitrate and 8-isoprostane concentrations were measured. Changes in body composition, REE, and systolic and diastolic BP were similar between the two interventions (*p* > 0.05). The CR + BRJ intervention produced greater changes in average microvascular flux (*p* = 0.03), NO-dependent endothelial activity (*p* = 0.02), and TMT-B cognitive scores (*p* = 0.012) compared to CR alone. Changes in urinary 8-isoprostane were greater in the CR + BRJ group (*p* = 0.02), and they were inversely associated with changes in average microvascular flux (r = −0.53, *p* = 0.003). These preliminary findings suggest that greater effects on vascular and cognitive functions could be achieved by combining CR with dietary nitrate supplementation.

## 1. Introduction

Obesity is a complex condition characterised by excessive body fat accumulation linked to the onset of serious comorbidities and increased risk of mortality [1,2,3]. Obesity-related complications include diabetes, hypertension, coronary heart disease, and musculoskeletal disorders [4,5,6]. Obesity has been linked to increased production of free radicals, sub-chronic inflammation, and reduced nitric oxide (NO) production, which represent key steps in the pathogenesis of metabolic, vascular, and neurodegenerative diseases [2,3,7,8,9,10]. Endothelial dysfunction is a key early pathogenetic step in the atherosclerotic process, and it is characterised by a reduction in the bioavailability of NO and loss or dysregulation of homeostatic mechanisms that operate in healthy endothelial cells [11,12].

Caloric restriction (CR) is a dietary strategy that decreases calorie intake without leading to malnutrition [13], which could help in losing body weight, especially FM reduction [14,15,16,17]. In addition, CR could increase the life expectancy [13,18,19,20,21,22,23,24,25,26] and improve vascular [19,21] and cognitive [20,27] functions; these effects may be mediated by a decrease in oxidative stress and inflammation [27,28,29,30] with consequent enhancement of mitochondrial efficiency, insulin sensitivity [22,31], and NO generation [32].

Dietary nitrate is a promising intervention that has been associated with improvement in metabolic [33,34,35], vascular (endothelial function and BP) [33,34,35,36], and, less consistently, brain (cognitive function, cerebral blood flow, and oxygenation) [37,38,39,40,41] functions. Nitrate-rich foods include spinach, lettuce, rocket, cabbage, and beetroot [42,43]. These positive effects are highly regulated by the induced production and bioavailability of NO via the non-enzymatic pathway (nitrate-nitrite-NO) [33,34,35,36,37,38,39,40,41]. The biological effector of dietary nitrate supplementation is NO, which is recognised for a wide range of cardiovascular and metabolic actions mediated by modulation of reactive oxygen species (ROS) production and degradation, immune response, endothelial function, insulin activity, and mitochondrial biogenesis and efficiency [34].

We postulate that CR and dietary nitrate could have synergistic effects on metabolic, vascular, and cognitive functions via their effects on common mechanistic pathways involving up-regulation of metabolic, endothelial, and neuronal functions. The aim of this pilot study was to investigate the impact of combining dietary nitrate and CR on metabolic (i.e., resting energy expenditure (REE), body composition, muscular strength) vascular (i.e., resting blood pressure and microvascular blood flow), and cognitive functions in overweight and obese middle-aged and older subjects.

## 2. Materials and Methods

### 2.1. Study Design

This was a two-arm, open-label, parallel randomised clinical trial conducted between February 2016 and December 2017 at the Nutrition and Dietetics facilities of the Faculty of Medical School of the University of Federico II of Naples, Italy. Laboratory analysis was completed in December 2018 at the University of Newcastle. Subjects were randomised into either a CR-alone (control group) or CR-plus nitrate-rich beetroot juice (CR + BRJ) intervention groups. This single-centre study was approved by the Ethics Committee of the Faculty of Medicine of the University Federico II of Naples, Italy (Approval No.: 8615). All subjects signed informed consent before participating in the study. The protocol was registered retrospectively with the ISCTRN database (ISRCTN59029976).

### 2.2. Participants

The study was conducted on 36 (18 per arm) overweight and obese (BMI: 25–40 kg/m^2^) middle-aged and older (50–75 years) men and women attending the research facilities. Participants were non-smokers and had an overall stable body weight in the last three months. A list of the inclusion and exclusion criteria for the selection of the participants is provided in the online Supplementary Material.

### 2.3. Nutritional Interventions

The CR intervention was based on a hypocaloric low-fat diet, which was prescribed to participants in both groups by a nutritionist. The REE was calculated using Fredrix’s equation and multiplied by a PAL of 1.5 to estimate total energy requirements [44]. The prescribed energy deficit was 40% of the total energy requirements, and the macronutrient composition was approximately CHO = 55–60%, FAT = 20–25%, and PRO = 15–20%. Participants were asked to maintain their habitual physical activity level and their consumption of alcoholic and caffeinated beverages. Participants in CR + BRJ group were asked to drink 70 mL of concentrated beetroot juice (~400 mg of inorganic nitrate daily) every morning (Beet It, James White Ltd., Ashbocking, Suffolk, UK). Participants were provided with a diary to record the time of the consumption and any problems that may have experienced. Participants were considered non-compliant if they did not take the supplementation on two or more days.

### 2.4. Study Protocol

Participants arrived in the morning for their first assessment visit. The study was conducted by a team of clinicians and nutritionists. The aim of the study was explained to eligible participants by a medically qualified investigator, and if the participants agreed, they signed the informed consent. Anthropometric (weight, height) and BP measurements were taken to confirm eligibility. Total energy requirements were determined to calculate the 40% caloric-restricted diet. Subjects were then randomised into one of the two interventions (CR + BRJ or CR alone).

After seven days, participants returned to the research centre to obtain information on the dietary and lifestyle intervention and to perform the baseline measurements. Participants arrived early in the morning after fasting for at least 8 h, provided urine and saliva samples, and then their REE was measured by indirect calorimetry. This was followed by measurements of endothelial function and resting BP. Next, bioelectrical impedance assessed body composition, and readings of bilateral hand-grip strength were taken. Subjects then completed a series of questionnaires, including an assessment of cognitive function, dietary intake, and physical activity level. After that, participants were provided with details about the CR interventions and, if allocated to the beetroot group, they were given 14 bottles of beetroot juice. The subjects started the intervention the day after the visit and continued for 14 days. On the 15th day, subjects arrived early in the morning fasting, and measurements were repeated in the same order as at the baseline visit. A description of the study protocol is provided in Figure S1 of the online Supplementary Material.

### 2.5. Body Composition

Weight was measured to the nearest 1 g using a standard beam scale (Seca GmbH & Co KG, Hamburg, Germany). Height was measured using a wall-mounted stadiometer to the nearest 0.1 cm. Waist circumference (WC) was measured at the midpoint between the last rib and the iliac crest on the midaxillary line. Bioelectrical impedance analysis (BIA) was undertaken by a single frequency (50 kHz) tetrapolar device (RJL 101; Akern SRL, Florence, Italy). BIA was performed with a single-frequency measurement. FM and FFM were obtained from measures of resistance and reactance using the algorithm provided by the manufacturer.

### 2.6. Indirect Calorimetry

REE was measured using indirect calorimetry (V MAX 29n, Sensor Medics, Yorba Linda, CA, USA). The device was calibrated before each measurement. Measurements were performed between 8:00 and 9:00 a.m. in a quiet, temperature-controlled room (22 ± 3 °C). The readings were analysed to see if the subject reached a steady state condition, indicated by the stability of oxygen and carbon dioxide volumes for at least five minutes. The metabolic test lasted between 25 min and 45 min. REE was calculated according to Weir’s Equation (7). Values of REE were presented unadjusted (kcal/day) and adjusted for FFM (kcal/day/kg).

### 2.7. Resting Blood Pressure

Resting SBP and DBP was measured in triplicate using an automated BP monitor (OMRON M3, OMRON Healthcare Europe, LR Hoofddorp, The Netherlands) after the participant had rested for at least 15 min. The average of the three measurements was calculated, and the obtained value was entered into the analysis.

### 2.8. Endothelial Function

All measurements were undertaken in a temperature-controlled room (22 ± 3 °C). Skin microvascular blood flow (SBF) was measured using a laser Doppler perfusion monitoring apparatus (PeriFlux 5000 System, Perimed, Stockholm, Sweden). The laser Doppler probe (PF 457, Perimed, Stockholm, Sweden), connected to a computer, was placed on the right forearm volar surface. After 10 min of acclimatisation, the blood flow was recorded for 20 min by Perisoft software. The mean skin blood perfusion was expressed as arbitrary perfusion units (PU). Next, for the measurement of post-occlusive reactive hyperemia, the brachial artery was occluded by a BP cuff placed on the right upper arm and inflated up to 50 mmHg above the SBP. The BP cuff was rapidly deflated after 3 min of brachial artery occlusion, and the peak value, expressed as PU, was determined by calculating the maximal perfusion value reached during reactive hyperemia.

### 2.9. Spectral Analysis

Microvascular blood flow oscillations in the range of 0.005 to 2.0 Hz were evaluated by Wavelet transform, a scale-dependent method comprising an adjustable window length able to analyse both low and high frequencies [45]. Spectral analysis was performed on the 20 min recordings under resting conditions to obtain a higher resolution of very low-frequency components. Wavelet analysis, proposed by Morlet, permits the detection of at least six frequency components in this interval, as reported by Kvandal et al. [46]. The contribution of endothelial NO-dependent and NO-independent blood flow was determined, and the obtained value was entered into the analysis.

### 2.10. Hand-Grip Strength

HGS was measured on the dominant and non-dominant hands to the nearest kilogram using a hand dynamometer (78010; Lafayette Instrument Company, Lafayette, IN, USA). During the measurement, the participant was in an upright position, and the arm of the measured hand was unsupported and parallel to the body. Three measurements were performed for the non-dominant hand, and the average of recorded measurements was used for the analysis.

### 2.11. Dietary Intake, Physical Activity, and Cognition Assessment

Dietary intake was assessed using a semi-quantitative food questionnaire based on the European Prospective Investigation into Cancer and Nutrition (EPIC) FFQ [47]. Physical activity was evaluated using the Italian version of the International Physical Activity Questionnaire—Short Form (IPAQ-SF) [48]. The Trail-Making Test (TMT) was administered to provide information on visual search, speed of processing, mental flexibility, and executive functions. The TMT consists of parts A and B. TMT-A requires an individual to draw lines sequentially connecting 25 encircled numbers distributed on a sheet of paper. Task requirements are similar for TMT-B, except the person has to alternate between numbers and letters in ascending order (e.g., 1, A, 2, B, 3, C, etc.). The score on each part represents the amount of time required to complete the task [49].

### 2.12. Urine and Saliva Collection

A spot urine sample was collected in the morning at the beginning and end of the intervention to measure nitrate concentrations. Stimulated saliva samples were collected using validated sample collection kits (Salivette, Sarsted, Germany). Samples were stored at −80 °C until analysis.

### 2.13. Compliance

The compliance with the dietary interventions was assessed by monitoring the dietary intake at baseline, after seven days (telephone interview), and at the end of the study. Compliance with nitrate supplementation was evaluated by completing a daily questionnaire and evaluating the changes in urinary nitrate concentration from baseline to the end of the study.

### 2.14. Laboratory Analysis

Nitrate and Nitrite: The ozone-based chemiluminescence method was used to measure plasma and salivary nitrate and nitrite concentrations, and urinary nitrate concentrations using the Sievers gas-phase chemiluminescence nitric oxide analyser (NOA 280i, Analytix), which has been described elsewhere [50].

Urinary 8-isoprostane: An ELISA Kit was used to analyse the amount of 8-isoprostane in the urine samples as a measure of oxidative stress (abcam UK, Cambridge, UK). The kit was a competitive immuno-enzymatic assay for quantitatively measuring 8-isoprostane in biological samples. Urine samples were diluted 4-fold prior to analysis. The assay’s sensitivity and precision (CV%) were 1 pg/mL and 1.75%, respectively.

### 2.15. Sample Size and Randomisation

The sample size calculation was performed using G-Power (version 3). The model selected for the study was a *t*-test model for independent measures. The outcome of the sample size calculation was the difference in REE between intervention and control. The difference between intervention and control was set to 150 kcal/day (SD: ±150 kcal/day). Power and significance levels were set at 0.80 and 0.05, respectively. Using these parameters, we estimated a total sample size of 18 participants per group, i.e., 36 participants in total. Randomisation was performed using an online service (www.envelope.com, accessed on 1 October 2015). Randomisation was run in blocks of 6 and letters (A and B) which were allocated to each intervention by a member of staff not involved in the study.

### 2.16. Statistical Analysis

Data were analysed using IBM SPSS Statistics (version 28.0). The normality of variables was checked visually by evaluation of the histograms and Shapiro—Wilks test. Descriptive statistics include mean, SD, or SE for continuous variables, and percentages (%) for the categorical variables. An independent *t*-test and chi-square tests were used to evaluate the baseline difference between the two intervention groups. Repeated-measure ANOVA was used to evaluate differences in physiological responses to the two interventions. Time (baseline and end values, T) was entered as a within-subject factor, and intervention groups (CR + BRJ or CR alone, I) as a between-subject factor. The interaction of the two terms (T*I) was evaluated for differences between the two interventions over time. The percent changes (Δ%) from the baseline for the primary and secondary outcomes were calculated, and an independent t-test was used to evaluate differences between groups. Pearson’s correlation was used to evaluate the changes in biomarkers’ concentrations (i.e., nitrate, nitrite, and 8-isoprostane) with physiological outcomes.

## 3. Results

### 3.1. Participants

**Figure 1:**
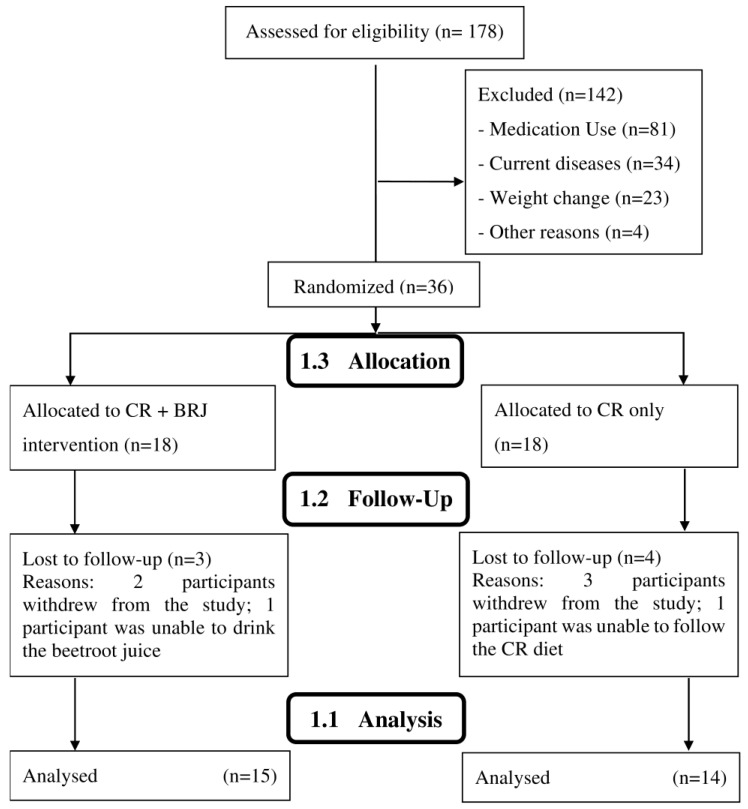
Flowchart showing the number of participants from the enrolment to the analysis. CR, caloric restriction; BRJ, beetroot juice.

A total of 178 participants were approached, and 36 were eligible and included in the study (18 participants per arm). Seven participants could not complete the experiment (three in the CR + BRJ and four in the CR group). Thus, 29 participants (22 females) were included in the final analysis (Figure 1). The mean age was 61.3 ± 5.9 years, ranging from 52 to 74 years. The mean BMI was 34.5 ± 5.8 kg/m^2^. Seventeen participants were taking medications or nutritional supplements (*n* = 17); they were equally distributed between the CR + BRJ and CR intervention groups (64% vs. 53%, respectively, *p* = 0.55). There were no significant differences between the groups for all baseline characteristics. Baseline characteristics are presented in Table 1.

### 3.2. Body Composition, Hand-Grip Strength, and Resting Energy Expenditure

Body weight decreased by −2.8 ± 1.7 kg (*p* < 0.001) in the CR + BRJ and −2.2 ± 1.2 kg (*p* < 0.001) in the CR alone group, but changes in body weight were not significantly different between groups (*p* = 0.32). Similarly, changes in FM (−1.3 ± 2.1 kg in CR + BRJ vs. −1.2 ± 2.1 kg in CR) and FFM (−1.4 ± 2.0 kg in CR + BRJ vs. −1.0 ± 2.3 kg in CR) in each group were significant (*p* = 0.003 and *p* = 0.006, respectively), but there was no significant difference between the two groups for both FM and FFM. HGS improved significantly in both groups (*p* < 0.001), and the percentage increase from the baseline was significantly higher in the CR + BRJ group (*p* = 0.04). The CR + BRJ and CR groups showed a similar decrease in REE (−26.5 ± 133.8 kcal/day and −42.4 ± 93.4 kcal/day, respectively, *p* = 0.71) post-weight loss. Post-weight loss changes in REE/FFM were not significantly different between the two groups (*p* = 0.66) (Table 2).

### 3.3. Vascular Function

SBP and DBP were significantly improved in CR + BRJ (−7.9 ± 6.7 and −2.9 ± 4.2 mmHg, respectively) and CR (−3.5 ± 5.9 and −1.6 ± 5.1 mmHg, respectively) (*p* < 0.01 for all), and there was a trend towards greater reductions in SBP in CR + BRJ compared with CR alone (*p* = 0.06). The average flux increased significantly in the CR + BRJ group compared to CR alone (+1.3 ± 2.4 PU vs. −1.2 ± 1.3 PU, respectively: T*I, *p* = 0.03). The NO-dependent endothelial activity derived from the spectral analysis was significantly increased in the CR + BRJ group (+7.0 ± 6.8%, T*I, *p* = 0.02) (Table 3).

### 3.4. Cognitive Function

TMT-A improved significantly (T, *p* < 0.005) in all groups but there was no significant difference between the two intervention groups (T*I, *p* = 0.54). The time to completion of the TMT-B was significantly reduced by the interventions (T, *p* = 0.002) with greater improvements observed in the CR + BRJ (−12.6 ± 6.8 s) compared to CR alone (−4.3 ± 9.7 s) (T*I, *p* = 0.012; Table 4).

### 3.5. Biomarkers

**Figure 2:**
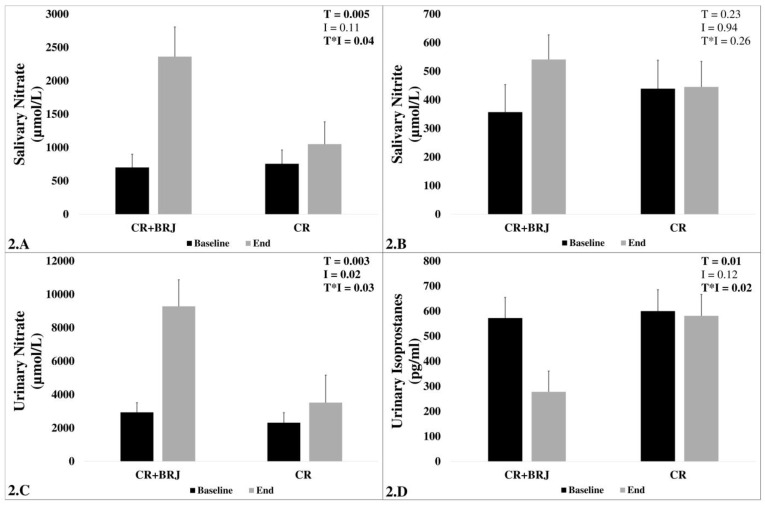
The effect of the interventions (CR + BRJ and CR) on the concentrations of salivary nitrate (**A**), salivary nitrite (**B**), urinary nitrate (**C**), and salivary isoprostanes (**D**) at the two-time points (baseline and end) among overweight and obese, middle-aged and older adults. Key: CI, confidence interval; CR, caloric restriction; CR + BRJ, caloric restriction with beetroot juice; I, intervention; *p*, *p*-value; T, time; T*I, time*intervention.

Salivary nitrate concentrations increased significantly by 1660 ± 1520 µmol/L in the CR + BRJ and by 294 ± 1879 µmol/L in the CR group (T, *p* < 0.005), with significant difference between the groups (T*I, *p* = 0.04, Figure 2A). Salivary nitrite concentrations were higher in the CR + BRJ group but did not reach statistical significance (T*I, *p* = 0.26, (Figure 2B). Changes in urinary nitrate concentrations were significantly higher in the CR + BRJ (+6344 ± 6233 µmol/L) compared to CR alone (+1206 ± 6012 µmol/L, T*I, *p* = 0.03; Figure 2C). Similarly, a greater decrease in urinary 8-isoprostane was observed in the combined CR + BRJ intervention (−294 ± 277 pg/mL, T*I, *p* = 0.02; Figure 2D).

### 3.6. Correlations

**Figure 3:**
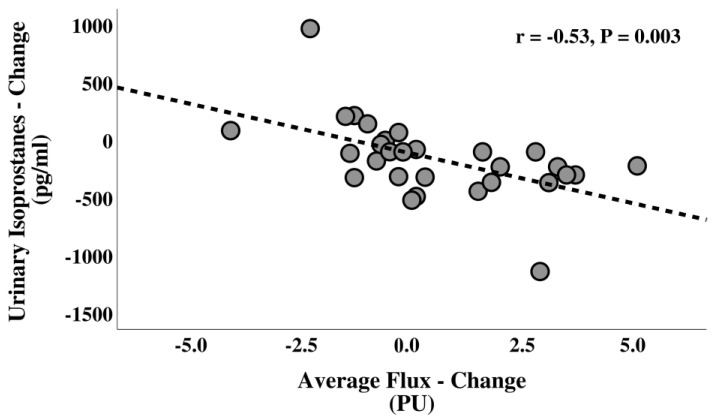
Correlation between changes in the urinary isoprostanes and average microvascular flux in overweight and obese, middle-aged and older adults. Key: *p*, *p* value; PU, perfusion unit; r, Pearson’s coefficient of correlation.

Overall, there were non-statistically significant correlations between the salivary nitrate and nitrite, as well as urinary nitrate and 8-isoprostane and metabolic, vascular, and cognitive outcomes. However, a significant negative correlation between urinary 8-isoprostane and average microvascular flux (r = −0.53, *p* = 0.003) was observed (Figure 3). Moreover, there was no modification of the association between 8-isoprostane and average flux after adjustment for changes in body weight (r = −0.53, *p* = 0.003).

## 4. Discussion

The results of this pilot study indicate the potential synergy between CR and dietary nitrate interventions and measures of vascular and cognitive functions without amplification of the adaptive effects following weight loss on energy metabolism. The study also indicates that the effects on endothelial function may be mediated by a reduction in oxidative stress.

A key finding of the study is that similar weight reduction and body composition changes occurred in both interventions; these were accompanied by the same extent of the decline in REE as a result of weight loss triggering adaptive metabolic mechanisms. Dietary nitrate supplementation has been associated with increased efficiency of mitochondria in skeletal muscle, which is one of the mechanisms underpinning the ergogenic effects of dietary nitrate [51]. The increased mitochondrial coupling produced a higher P:O ratio but also a reduction in heat dissipation [51]. The macroscopic effect of these mechanisms was tested in a 3-day dietary nitrate supplementation trial showing a reduction of REE by 4.2% (−82 kcal/day) measured by indirect calorimetry in healthy subjects [52]. However, these initial findings have not been replicated in subsequent studies of different duration (i.e., 2 h or 7 days) as no significant changes were found for REE after nitrate supplementation [53,54]. All these studies were conducted in energy balance whereas our study purposively investigated whether dietary nitrate may amplify the expected (i.e., due to the decrease in metabolically active tissue) reduction in REE post-weight loss induced by CR. The similar decrease in REE in the two intervention groups is reassuring as dietary nitrate does not seem to amplify the initial adaptative metabolic responses to caloric restriction, and no additional adjustments in energy intake may be needed to limit the risk of weight regain.

Both CR and dietary nitrate supplementation have been independently linked to improved BP and endothelial function in several studies [36,55], but the synergy of the combination of the two interventions has not been tested so far. This study corroborates these positive effects, but it also shows the potential existence of a synergy between the two interventions as an almost statistically significant greater drop in systolic BP was observed in the CR + BRJ group, which also exhibited greater improvements in endothelial function (average flux and NO-dependent endothelial activity). The vascular effects could be explained by the enhancement of endothelial function mediated by various mechanisms, such as (i) increased NO production through SIRT1/AMPK/Akt-eNOS-NO pathway [32,56,57,58,59] and nitrate-nitrite-NO pathway via dietary nitrate [33,34,35], (ii) greater insulin sensitivity [22,59,60,61,62,63,64], (iii) upregulation of antioxidants defenses [27,28,29,30,59,65,66,67] and reduced ROS generation via increased mitochondrial efficiency.

The promising effects on ROS production were supported by a significantly greater reduction in urinary 8-isoprostane concentrations in the CR + BRJ group, and a significant inverse correlation between isoprostanes and average microvascular flux was observed. Isoprostanes are prostaglandin-like compounds and are commonly used as biomarkers of oxidative stress and increased levels have been associated with a greater risk for cardiovascular and neurodegenerative diseases in animal and human experiments [68,69]. This result must e interpreted with caution as betanins present in beetroot may have antioxidant properties and contribute to the reduction in isoprostanes concentrations [70,71].

Both interventions showed an overall improvement in cognitive function, but changes in the TMT-B scores were only significant in the CR + BRJ group. Justice et al. [72] showed a similar result in healthy older subjects as time to complete TMT-B was improved by 18% and 14% in response to supplementation with high and low doses of sodium nitrite for 10 weeks, respectively. Potential mechanisms explaining the cognitive effects could include a CR-induced Akt phosphorylation through the insulin-PI3K-Akt signalling pathway [73,74], increased NO production, and potentiation of pre-synaptic neurotransmission [75,76] occurring alongside an enhancement of neurovascular and metabolic coupling [77].

Babateen et al. [78] showed that different doses of beetroot supplementation for 13 weeks did not improve cognitive function and CBF. Therefore, the promising effects of dietary nitrate and CR on cognition need confirmation in larger and longer studies employing sophisticated methods (i.e., computerized cognitive assessment tools and imaging methods) to corroborate the results observed in this study.

### Strengths and Limitations

This study is characterised by some limitations that need to be taken into account in the interpretation of the results. First, the sample size and duration are important limitations. However, this is the first pilot study to test the physiological effects of a concomitant CR and dietary nitrate intervention. Previous RCTs investigating the effects of either CR or dietary nitrate had similar sample sizes and study duration [36,79]. Some of the participants were taking supplements, or medications (such as antihypertensive, antidyslipidemic, antidepressant, and thyroxine), which may have influenced some of the results. However, the doses of the drugs were stable prior to and during the study, and the distribution of drugs and supplements among participants was not significantly different between the two intervention groups. The unblinded design and the lack of a control group for the dietary nitrate intervention (i.e., nitrate-depleted beetroot juice) are additional limitations, and these factors should be considered in the design of future studies to test the combined effects of CR and dietary nitrate. Urinary nitrate, nitrite, and isoprostane concentrations were not adjusted for potential differences in renal function as 24 h total urine volume or serum and urinary creatinine concentrations were not measured. Epidemiological studies have suggested a potential carcinogenic association with dietary nitrate [80,81]. However, the evidence of the association of dietary nitrate intake with cancer risk is still mixed [82], which may be confounding by the consumption of processed meats containing nitrates and nitrites used as food preservatives [82]. The current WHO acceptable and recommended daily intake for nitrate is 3.7 mg/kg/day or lower, which approximately ranges from 260 mg/day to 370 mg/day of nitrate in individuals with a body weight between 70 and 100 kg. The daily dose of nitrate provided to the participants in this study was higher than ADI recommendations but it was also considerably lower than nitrate doses provided in several previous studies reporting beneficial effects on cardiovascular outcomes [36,55]. Hord et al. have also calculated the nitrate content of healthy dietary patterns, such as the Mediterranean diet or DASH diet, which could contain up to 1000 mg of dietary nitrate [43].

## 5. Conclusions

This pilot RCT showed that the supplementation of dietary nitrate alongside a calorically restricted diet could be a promising strategy to improve vascular and cognitive functions in older overweight subjects. Dietary nitrate does not appear to amplify the drop in energy metabolism occurring with weight loss. Further investigations in studies with more robust designs and larger study populations are warranted to substantiate these promising initial findings.

## Figures and Tables

**Table 1:** Baseline characteristics.

	Total (*n* = 29)	CR + BRJ (*n* = 15)	CR (*n* = 14)	*p*
Age, y	61.3 ± 5.9	59.5 ± 5.9	63.2 ± 5.3	0.08
Female, n (%)	22 (75.9)	11 (73.3)	11 (78.5)	0.74
Weight, kg	89.0 ± 19.1	91.7 ± 19.6	86.1 ± 18.8	0.44
Height, cm	160.1 ± 10.3	161.5 ± 12.7	158.6 ± 7.0	0.46
BMI, kg/m^2^	34.5 ± 5.8	34.8 ± 4.4	34.1 ± 7.2	0.74
WC, cm	104.9 ± 13.2	104.6 ± 12.6	105.3 ± 14.2	0.88
FM, kg	35.2 ± 10.6	35.8 ± 9.5	34.5 ± 12.0	0.75
FFM, kg	53.7 ± 13.1	55.8 ± 15.6	51.5 ± 9.9	0.38
HGS, kg	25.7 ± 10.2	26.7 ± 12.2	24.7 ± 7.8	0.61
IPAQ, METs/w	3401 ± 4470 *	4030 ± 5319	2727 ± 3408	0.44
EI, kcal/d	1448 ± 165	1460 ± 183	1434 ± 149	0.68

The values of the continuous variables were presented as mean and standard deviation, whereas the categorical variable (gender) was presented as percentages. * Non-normal distribution (median and IQR, tested by Mann—Whitney test). Key: BMI, body mass index; CR, caloric restriction; CR + BRJ, caloric restriction with beetroot juice; EI, energy intake; FFM, fat-free mass; FM, fat mass; HSG, hand-grip strength; IPAQ, international physical activity questionnaire; IQR, interquartile range; *p*, *p*-value; WC, waist circumference.

**Table 2:** Anthropometry, body composition, hand-grip strength, and resting energy expenditure.

Variable	CR + BRJ			CR			*p*
	Baseline	End	∆%	Baseline	End	∆%	
Weight, kg	91.7 ± 19.6	88.9 ± 18.1	−2.8 ± 1.2	86.1 ± 18.8	83.8 ± 18.0	−2.5 ± 1.1	T < 0.001 I = 0.44 T*I = 0.32 ∆% = 0.44
WC, cm	104.6 ± 12.6	101.0 ± 12.5	−3.4 ± 2.2	105.3 ± 14.2	102.5 ± 13.3	−2.6 ± 1.7	T < 0.001 I = 0.82 T*I = 0.33 ∆% = 0.27
FM, kg	35.8 ± 9.5	34.5 ± 10.2	−4.4 ± 7.9	34.5 ± 12.0	33.3 ± 12.3	−3.7 ± 7.2	T = 0.003 I = 0.76 T*I = 0.91 ∆% = 0.8
FFM, kg	55.8 ± 15.6	54.4 ± 15.7	−2.7 ± 3.5	51.5 ± 9.9	50.5 ± 9.2	−1.6 ± 4.3	T = 0.006 I = 0.4 T*I = 0.56 ∆% = 0.47
HGS, kg	26.7 ± 12.2	29.7 ± 10.2	18.4 ± 25.0	24.7 ± 7.8	25.4 ± 7.1	4.0 ± 7.4	T < 0.001 I = 0.38 T*I = 0.34 ∆% = 0.04
REE, kcal/d	1438 ± 222	1411 ± 263	−1.8 ± 10.3	1355 ± 216	1313 ± 213	−2.9 ± 6.7	T = 0.12 I = 0.28 T*I = 0.71 ∆% = 0.74
REE/FFM, kcal/d/kg	26.6 ± 4.2	26.6 ± 3.4	0.8 ± 9.9	26.5 ± 2.4	26.1 ± 1.8	−1.1 ± 8.3	T = 0.63 I = 0.79 T*I = 0.66 ∆% = 0.56

Repeated measure ANOVA was used to evaluate the effect of the interventions on the selected outcomes. An independent T-test was used to compare the difference in the percent change. Key: CR, caloric restriction; CR + BRJ, caloric restriction with beetroot juice; FFM, fat-free mass; FM, fat mass; HSG, hand-grip strength; I, intervention; *p*, *p*-value; REE, resting energy expenditure; REE/FFM, resting energy expenditure/fat-free mass; T, time; T*I, time*intervention; WC, waist circumference; ∆%, change percentage.

**Table 3:** Vascular function.

Variable	CR + BRJ			CR			*p*
	Baseline	End	∆%	Baseline	End	∆%	
SBP, mmHg	131.2 ± 6.1	123.2 ± 6.0	−5.9 ± 4.9	129.0 ± 9.0	125.5 ± 8.4	−2.6 ± 4.4	T < 0.001 I = 0.97 T*I = 0.06 ∆% = 0.06
DBP, mmHg	81.0 ± 8.0	78.1 ± 6.2	−3.2 ± 4.8	79.1 ± 7.3	77.5 ± 7.7	−1.8 ± 6.0	T < 0.01 I = 0.64 T*I = 0.46 ∆% = 0.50
Average flux, PU	7.2 ± 3.0	8.5 ± 2.5	26.1 ± 35.8	7.6 ± 2.1	7.3 ± 1.8	−2.2 ± 21.2	T = 0.18 I = 0.57 T*I = 0.03 ∆% < 0.01
Peak flux, PU	54.4 ± 10.8	58.1 ± 11.2	7.6 ± 13.7	46.6 ± 15.6	50.4 ± 22.3	9.2 ± 43.6	T = 0.13 I = 0.15 T*I = 0.99 ∆% = 0.89
NO-dependent EA, %	10.5 ± 4.7	17.4 ± 3.7	95.4 ± 96.3	10.4 ± 4.6	11.7 ± 5.0	24.9 ± 67.1	T < 0.001 I = 0.02 T*I = 0.02 ∆% = 0.03
NO-independent EA, %	2.2 ± 1.1	1.9 ± 1.6	10.7 ± 145.3	3.0 ± 2.0	3.5 ± 2.4	63.2 ± 161.3	T = 0.86 I = 0.03 T*I = 0.36 ∆% = 0.36

Repeated measure ANOVA was used to evaluate the effect of the interventions on the selected outcomes. An independent T-test was used to compare the difference in the change percentage. Key: CR, caloric restriction; CR + BRJ, caloric restriction with beetroot juice; DBP, diastolic blood pressure; EA, endothelial activity; I, intervention; NO, nitric oxide; PU, perfusion unit; *p*, *p*-value; SBP, systolic blood pressure; T, time; T*I, time*intervention; ∆%, change percentage.

**Table 4:** Cognitive function.

Variable	CR + BRJ			CR			*p*
	Baseline	End	∆%	Baseline	End	∆%	
TMT-A, s	40.8 ± 19.2	34.4 ± 12.3	−13.5 ± 12.6	49.2 ± 23.3	44.9 ± 17.5	−3.6 ± 19.9	T = 0.005 I = 0.16 T*I = 0.54 ∆% = 0.12
TMT-B, s	94.1 ± 19.5	81.5 ± 19.7	−13.6 ± 8.7	95.8 ± 33.4	91.5 ± 30.7	−3.2 ± 11.6	T < 0.001 I = 0.54 T*I = 0.012 ∆% = 0.011

Repeated measure ANOVA was used to evaluate the effect of the interventions on the selected outcomes. An independent T-test was used to compare the difference in the change percentage. Key: CR, caloric restriction; CR + BRJ, caloric restriction with beetroot juice; I, intervention; *p*, *p*-value; T, time; T*I, time*intervention; TMT-A, trail making test part a; TMT-B, trail making test part b; ∆%, change percentage.

## Data Availability

Not applicable.

## Supplementary Materials

The following supporting information can be downloaded at: https://www.mdpi.com/article/10.3390/nu15040890/s1, Figure S1: Study Protocol; Table S1: Inclusion and exclusion criteria.
