# Supplementary material for: Caloric Restriction (CR) Plus High-Nitrate Beetroot Juice Does Not Amplify CR-Induced Metabolic Adaptation and Improves Vascular and Cognitive Functions in Overweight Adults: A 14-Day Pilot Randomised Trial
Source: Nutrients. 2023 Feb 10;15(4):890. doi: 10.3390/nu15040890 (PMC9962072; doi:10.3390/nu15040890)
Supplement: Supplementary file 1 [file nutrients-15-00890-s001.zip › nutrients-2197568-supplementary.pdf]

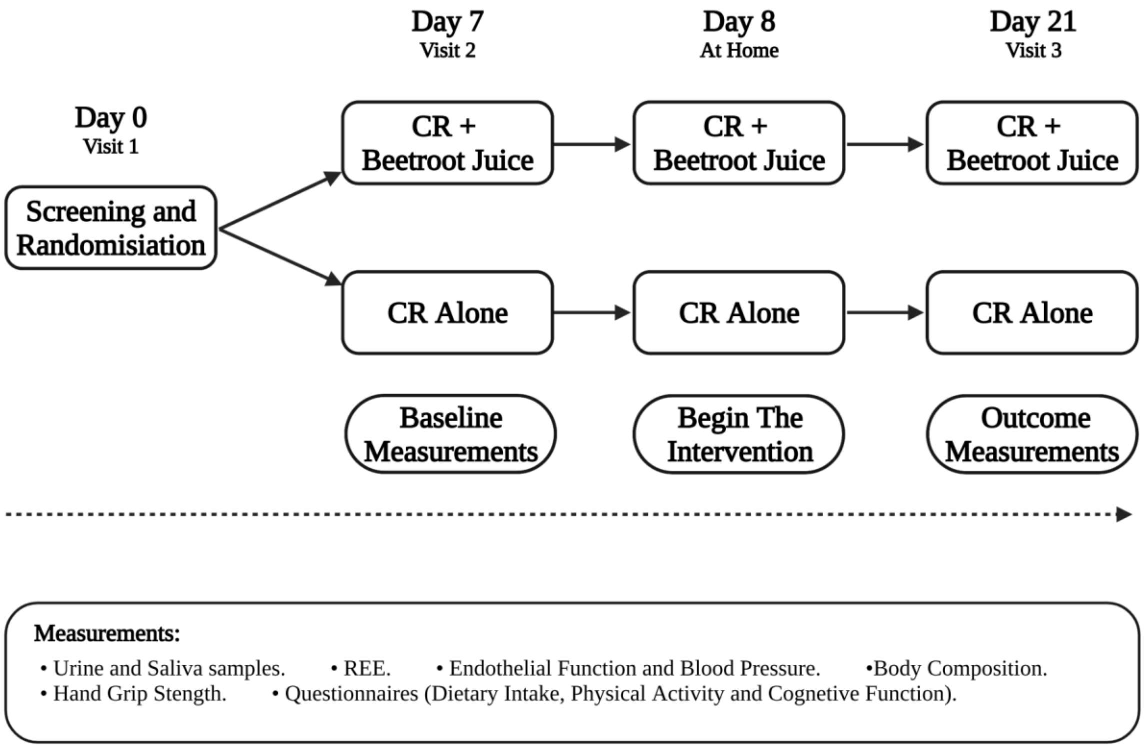


**Figure S1: Study protocol**

**Table S1: Inclusion and exclusion criteria**

**Inclusion criteria were:**
- Overweigth and Obesity (BMI Range 25-40 kg/m$^2$)
- Men and woman in the age range of 50 - 75 years
- Non-smoker
- Stable weight

**Exclusion criteria are (reason for exclusion):**
- Current participation in other research clinical studies
- Vegetarianism (likely to have very high nitrate intake)
- Weight change more than 3.0kg in the last 2 months (important influence on systemic metabolism and vascular function).
- Active cancer and any diagnosis of malignant cancer in the last 5 years (systemic effects on study outcomes).
- Diagnosis of chronic and acute metabolic and inflammatory conditions in-terfering with the study outcome (systemic effects on study outcomes). For example flu, Chrohn’s Disease, rheumatoid arthritis.
- Previous diagnosis of type 1 or type-2 diabetes treated with insulin (modification of regulation of intermediate metabo-lism). Type 2 diabetic patients treated with oral hypoglycaemic agents and/or diet only will be included in the study.
- Weight loss medications (sibutramine, orlistat, rimonabant) and history of bariatric surgery (weight loss related changes in systemic metabolism).
- Drugs: corticosteroids, sildenafil, aspirin, NSAIDs, diuretics, antacids, anticoagulants, nitrate-derived agents, anti-cholinergic, (all drugs may have either an effect on NO production or insulin sensitivity via differ-ent mechanisms).
- Subjects on hormonal therapies (oestrogens, thyroxine, progesteron), anti-hypertensive (Ca++ channel blockers, beta-blockers, ACE inhibitors), statins and any oth-er antidyslipidaemic agent, and psychiatric drugs (antidepressants, seda-tives, anti-psychotics) will be excluded if dose has been started/changed in the previous three months. (make sure that these disorders are under strict control to avoid interference with the study outcomes).
- Severe haematological (risk for the participant and effects on the study outcomes).
- Major surgical operations interfering with the study outcomes (systemic effects on study outcomes).
- Alcohol intake >21 units/week for men and >14 unites/week women
- Blood donations in the previous 3 months.
